# Terminal dribbling in male patients with lower urinary tract symptoms: relationship with International Prostate Symptom Score and with intravesical prostatic protrusion

**DOI:** 10.1186/s12894-015-0082-x

**Published:** 2015-08-29

**Authors:** Jae Heon Kim, Ji Sung Shim, Hoon Choi, Du Geon Moon, Jeong Gu Lee, Je Jong Kim, Jae Hyun Bae, Jae Young Park

**Affiliations:** Department of Urology, Soonchunhyang University Hospital, Soonchunhyang University College of Medicine, Seoul, Republic of Korea; Department of Urology, Korea University Ansan Hospital, Ansan, Republic of Korea; Department of Urology, Korea University College of Medicine, Seoul, Republic of Korea

## Abstract

**Background:**

Terminal dribbling is one of the lower urinary tract symptoms (LUTS) that has not been widely studied. The aim of this study was to investigate the associations between terminal dribbling (TD) and other parameters such as International Prostate Symptom Score (IPSS) and intravesical prostatic protrusion (IPP).

**Methods:**

Medical records of male patients with LUTS aged 40 years and older were prospectively collected. Data regarding TD defined by the International Continence Society standardization subcommittee, IPSS, prostate-specific antigen, total prostate volume, and IPP on transrectal ultrasonography were obtained. TD was confirmed by the subsequent uroflowmetry (uroflowmetry-confirmed TD). Logistic regression analysis was performed to identify the parameters affecting TD and uroflowmetry-confirmed TD.

**Results:**

Among the 578 men, 226 patients (39.1 %) complained of TD and 157 patients (27.2 %) had objective findings of TD on uroflowmetry. In the logistic regression analysis, IPSS voiding subscore were correlated with TD (Odds ratio 1.06). In addition, IPP was the only significant risk factor for uroflowmetry-confirmed TD (Odds ratio 2.83). Each question of IPSS is not correlated with TD or uroflowmetry-confirmed TD.

**Conclusions:**

While the symptom of TD is well correlated with IPSS voiding subscore, objective evidence of TD on uroflowmetry had strong correlation with IPP. TD should be investigated further to reveal its clinical impact and guide a proper management.

## Background

Terminal dribbling (TD) is known to be common in men with or without benign prostate hyperplasia (BPH), and has been shown to have a negative impact on quality of life [1–3]. TD has been included in the symptom score suggested by Boyarsky et al. and the International Continence Society (ICS) male questionnaire [4, 5]. In 1996, the study done by Abrams et al. pointed out that objective evidence of TD is significantly related with bladder outlet obstruction [6].

TD is newly defined by the ICS standardization subcommittee in 2002 [7]. However, no study has been carried out according to this definition up to now even though it is the most common symptom among lower urinary tract symptom (LUTS) in BPH patients or community people [4]. In the present study, we sought to investigate the prevalence of TD among LUTS patients and its relationship with intravesical prostatic protrusion (IPP) and with International Prostate Symptom Score (IPSS).

## Methods

### Study population

The study comprised 635 consecutive male LUTS patients aged 40 years and older attending as new patients at the outpatient clinic between January 2008 and July 2013. This study was conducted in accordance with the ethical standards laid down in the 1964 Declaration of Helsinki and its later amendments; appropriate ethical review boards (Korea University Medical Center Ansan Hospital IRB No. AS 14156) approved this retrospective study. The patients with LUTS mean that the patients who have storage symptoms, voiding symptoms or post micturition symptoms as described in the previous study [7]. Fifty seven of these men were excluded from our study due to biopsy proven prostate cancer, other malignancies, previous medication for LUTS, past history of surgery for BPH or other pelvic diseases, neurologic abnormalities, or missing data. IPSS, prostate-specific antigen (PSA), prostate volume, prostate transitional zone volume and IPP on transrectal ultrasonography (TRUS), and the answer to the TD item on the ICS male questionnaire were recorded for each subject.

### Terminal dribbling definition and confirmation

TD was defined in the ICS guidelines as “when an individual describes a prolonged final part of micturition when the flow has slowed to a trickle/dribble” [7]. The questionnaire item regarding TD was stated as follows: “Do you have a prolonged final part of micturition when the flow has slowed to a trickle/dribble?” Answer choices for this question included yes and no. Uroflowmetry-confirmed TD means that TD confirmed by the uroflowmetry when the typical finding of slowly decreasing flow with trickling/dribbling at the end of the void was observed.

### Features measured using transrectal ultrasonography

The prostate was measured in three dimensions on TRUS, and its volume and transitional volume were estimated using a modification of the prolate ellipsoid formula and expressed in cm3 (0.523 [length (cm) × width (cm) × height (cm)]) [8]. IPP was measured as the vertical distance from the tip of the protruding prostate to the base of the urinary bladder in the sagittal plane, and it was graded as mild (less than 5 mm), moderate (from 5 to less than 10 mm), severe (10 mm or more) [9, 21]. Urology residents performed this procedure under the supervision of attendings. We divided patients into two groups based on the presence of IPP when performing logistic regression analysis.

### Acquisition of IPSS questionnaires

The severity of LUTS was measured using the IPSS, which is based on the American Urological Association symptom index with one additional question on quality of life (QoL). After the patients were asked to complete the IPSS questionnaire, voiding subscore (sum of scores in 1st, 3rd, 5th, and 6th question of IPSS) and storage subscore (sum of scores in 2nd, 4th, and 7th question of IPSS) were calculated [10]. The Korean version of the IPSS was also validated in terms of relevance and reliability, and is now used as a diagnostic tool for LUTS [11].

### Statistical analysis

Continuous variables were expressed as either the mean ± standard deviation, or median [inter-quartile range]. Categorical variables were reported as the number of occurrences and frequency. Student t-test and the Pearson χ2 test were used for statistical comparisons of continuous and categorical variables, respectively. Simple and multiple logistic regressions with a backward variable selection procedure were performed to identify the parameters affecting TD. Simple logistic regressions were performed to reveal the association between TD and IPSS. Data were analyzed using PASW Statistics version 18.0 (SPSS, Inc. Chicago, IL, USA). We regarded a p value <0.05 as statistically significant.

## Results

### Characteristics of enrolled patents

**Fig. 1:**
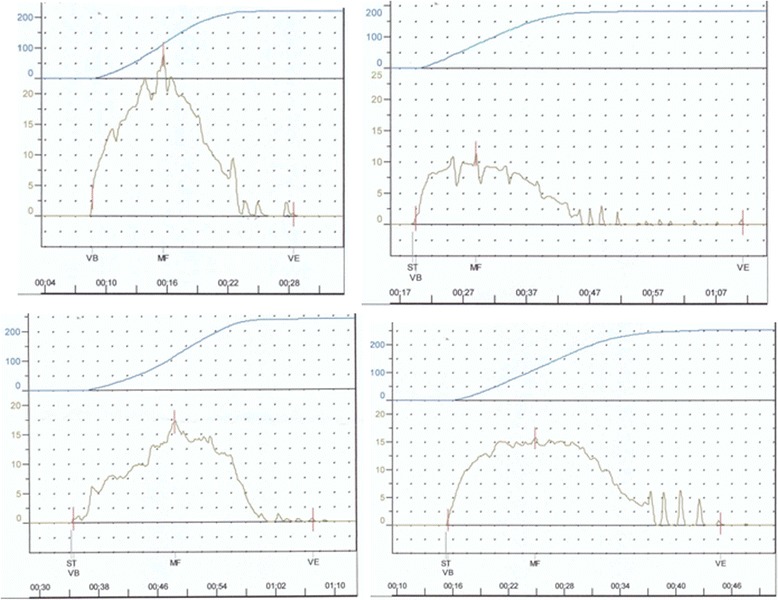
Typical findings of terminal dribbling on uroflowmetry

The mean age of subjects was 62.4 years, the median prostate volume was 31.0 cm^3^, and the median transitional volume was 12.0 cm^3^ (Table 1). Among 578 men with LUTS, 226 men (39.1 %) complained of TD. Among these 226 men who complained of TD, TD was confirmed in 157 men (uroflowmetry-confirmed TD group) by subsequent uroflowmetry. Typical findings such as slowly decreasing flow with trickling/dribbling at the end of the void were observed in these patients (Fig. 1). When the patients were classified with age, 23 men (10.1 %) in their 40’s complained of TD, 65 men (28.6 %) in their 50’s complained of TD, 81 men (35.7 %) in their 60’s, and 58 men (25.6 %) in their 70’s, respectively. There were no significant differences between TD and non-TD groups with respect to age, PSA, prostate volume or prostate transitional zone volume. However, IPSS total score, IPSS voiding subscale score, IPSS storge subscale score, and IPSS QoL score in TD group were significantly higher than those in non-TD group. Among 578 men with LUTS, IPP was observed in 248 patients (42.9 %). The number of patients with IPP was not significantly different in TD group than that in non-TD group (p value 0.402). When classified by uroflowmetry-confirmed TD, the number of patients with IPP was significantly higher in uroflowmetry-confirmed TD group (95 out of 157) than that in patients who did not show the typical finding of TD in uroflowmetry test (153 out of 421) (Odds ratio 2.67).

**Table 1:** Baseline characteristics of enrolled patients

Variables	All cases (*n* = 578)	Terminal dribbling (*n* = 226)	Non-Terminal dribbling (*n* = 352)	p value
Age (years), mean ± SD	62.4 ± 10.8	62.2 ± 10.3	62.6 ± 11.03	0.775
PSA (ng/ml), median [IQR]	2.22 [0.75-5.46]	1.70 [0.73-4.81]	2.65 [0.76-5.87]	0.098
Prostate volume (cm^3^), median [IQR]	31.0 [23.0-44.0]	29.0 [22.0-43.0]	32.0 [23.0-45.0]	0.261
Prostate transitional zone volume (cm^3^), median [IQR]	12.0 [7.0-20.0]	10.0 [6.0-18.0]	13.0 [7.25-22.0]	0.116
IPSS total score, Mean ± SD	14.1 ± 8.67	15.7 ± 8.33	13.1 ± 8.72	<0.001
Voiding subscale score, mean ± SD	8.13 ± 5.76	9.23 ± 5.44	7.43 ± 5.85	<0.001
Storage subscale score, mean ± SD	5.99 ± 3.70	6.49 ± 3.67	5.66 ± 3.66	0.008
IPSS QoL score, Mean ± SD	3.64 ± 1.15	3.83 ± 1.07	3.51 ± 1.20	0.002
IPP (%)	248 (42.9 %)	100 (44.2 %)	148 (40.2 %)	0.402
Mild (%)	362 (15.3 %)	12 (12.0 %)	26 (17.6 %)	
Moderate (%)	149 (60.1 %)	64 (64.0 %)	85 (57.4 %)	
Severe (%)	65 (24.6 %)	24 (24.0 %)	37 (25.0 %)	

IPSS = International Prostate Symptom Score; QoL = Quality of Life; IPP = intravesical prostatic protrusion

### The risk factors for terminal dribbling in the logistic regression analysis

In the logistic regression analysis with a backward variable selection procedure, voiding subscore was the only significant risk factor for TD in the enrolled patients (Odds ratio 1.06, Table 2). In addition, when the multiple logistic regression analysis was performed to reveal the significant factor for uroflowmetry-confirmed TD, IPP was the only significant risk factor (Odds ratio 2.83, Table 3). Each question of IPSS is not correlated with TD or uroflowmetry-confirmed TD in the logistic regression analysis.

**Table 2:** The logistic regression analysis identifying the factors affecting terminal dribbling

Variable	Logistic Regression		
	OR	95 % Confidence interval	p value
Age	1.00	0.98 – 1.02	0.707
PSA	0.98	0.94 – 1.02	0.262
Prostate volume (cm^3^)	1.00	0.99 – 1.01	0.626
Prostate transitional zone volume (cm^3^)	0.98	0.96 – 1.00	0.089
IPP	1.41	0.96 – 2.09	0.083
IPSS total	1.00	0.93 – 1.07	0.901
Voiding subscore	1.06	1.02 – 1.09	0.001
Storage subscore	0.96	0.88 – 1.05	0.384
IPSS QoL	1.15	0.94 – 1.41	0.170

OR = odds ratio; PSA = prostate-specific antigen; IPP = intravesical prostatic protrusion; IPSS = International Prostate Symptom Score; QoL = Quality of Life

*Backward variable selection procedure was applied

**Table 3:** The logistic regression analysis identifying the factors affecting uroflowmetry-confirmed terminal dribbling

Variable	Logistic Regression		
	OR	95 % Confidence interval	p value
Age	0.99	0.97 – 1.01	0.530
PSA	0.97	0.92 – 1.02	0.273
Prostate volume (cm^3^)	1.00	0.99 – 1.01	0.752
Prostate transitional zone volume (cm^3^)	1.00	0.99 – 1.02	0.713
IPP	2.83	1.91 – 4.21	<0.001
IPSS total	0.96	0.89 – 1.04	0.363
Voiding subscore	1.03	0.93 – 1.15	0.561
Storage subscore	1.01	0.96 – 1.07	0.600
IPSS QoL	1.23	0.98 – 1.55	0.070

OR = odds ratio; PSA = prostate-specific antigen; IPP = intravesical prostatic protrusion; IPSS = International Prostate Symptom Score; QoL = Quality of Life

*Backward variable selection procedure was applied

## Discussion

One of the difficulties encountered in earlier studies regarding TD was the lack of a clear definition ensuring that patients could discriminate it from post-void dribbling. The current concept of TD was first addressed on the ICS male questionnaire and has been defined by ICS recommendations in 2002 [4, 7]. Before this definition, the studies performed in 1990s used various definitions of TD [12–16]. Moreover, several reports have regarded TD as an initial or mild form of post-void dribbling [13–15]. Post-void dribbling was recently determined to be one of post-micturition symptoms that is distinct from TD. The pathogenesis of post-micturition dribbling involves weakness of the bulbocavernosus muscle, which results in failure to evacuate urine that has pooled and trapped in the bulbar area of the urethra after voiding [17]. Additionally, the ICS previously removed the term “terminal dribbling” because of its similarity with post-void dribbling, which could be the main reason why there have been few studies on this issue [18]. To achieve the consistency, we conducted face-to-face interview by a single investigator using standardized definition of TD. Clinical studies on this topic should describe the demographic characteristics of the subjects, the prevalence of TD, the questionnaire used, and the definition of TD. Table 4 summarizing the previous studies related with TD indicates that the prevalence of TD is much greater in the LUTS patients than in the community people.

**Table 4:** Summary of previous studies on terminal dribbling

Source	Study sample	Prevalence	Questionnaires used	Definition of terminal dribbling	Remark
Boyarsky *et al.* (1977) ^4^	Not a clinical study	Not determined	A pilot questionnaire with 10 items	No specific definition of terminal dribbling.	Suggested a guideline with a 10 item questionnaire including terminal dribbling.
Garraway *et al.* (1991)^20^	Community sample	45 %	A pilot questionnaire by Fowler^16^	Patients were asked to rate the frequency of dribbling after urination. (Confusion with post-void dribbling)	Uroflowmetry and transrectal ultrasonography were done. Undetermined impact of terminal dribbling
Meyhoff *et al.* (1993)^12^	LUTS patients	Not determined	DAN-PSS-1	“Do you experience dribbling after voiding, when you feel you have finished urination?” (Confusion with post-void dribbling)	Post-void dribbling was aggravated after transurethral prostatectomy.
Chute *et al.* (1993) ^13^	Community sample	36–44 %	A pilot questionnaire by Epstein^18^	“Dribbling after urinating.” (Confusion with post-void dribbling)	Uroflowmetry and transrectal ultrasonography were done. Terminal dribbling was noted to be bothersome.
Reynard *et al.* (1996)^6^	LUTS patients	44 % in questionnaire, 27 % in uroflowmetry	Not determined	“Does your urinary stream end with a dribble?” Gradient of a line drawn between the maximum flow rate and the end of flow was <0.25 and if, in the terminal 15 s of uninterrupted flow, the flow rate did not exceed 5 ml/s at any point.	Pressure-flow study was done. Terminal dribbling on questionnaire was not related to BOO defined by pressure-flow study.
Hughes *et al.* (2000)^1^	Community sample	35 %	ICS male questionnaire	“Do you have any trickle/dribble at the final part of micturition?”	Terminal dribbling was the single- most bothersome symptom.
Scarpa *et al.* (2001)^2^	LUTS patients	88 %	ICS male questionnaire	“Do you have any trickle/dribble at the final part of micturition?”	Terminal dribbling was the both most common and bothersome symptom
Jin *et al.* (2003)^3^	LUTS patients	85.6 %	ICS male questionnaire	“Do you have any trickle/dribble at the final part of micturition?”	Translated questionnaire in Korean. Pressure-flow study was done. IPP was not checked.
Yano *et al.* (2004)^16^	LUTS patients	Not determined	Saitama Prostate Symptom Score	“Do you experience dribbling after voiding, when you feel you have finished urination?”	Validation study with IPSS. Pressure-flow study was done. Undetermined impact of terminal dribbling.
Shiri *et al.* (2005)^14^	Community sample	52 %	DAN-PSS-1^20^	“Do you consider your urinary stream as dribbling?”	Relationship between LUTS and ED. Terminal dribbling was related to ED.

LUTS = lower urinary tract symptoms; ICS = International Continence Society; BOO = bladder outlet obstruction; IPSS = International Prostate Symptom Score; IPP = intraprostatic protrusion; DAN-PSS-1 = Danish Prostate Symptom Score; ED = erectile dysfunction

Although few studies have been perform to investigate TD, high rates have been observed in patients with LUTS in both Western and Asian populations [2, 4, 19]. The ICS–‘BPH’ study reported 91–96 % of symptom prevalence of TD in BPH patients and 73–79 % in community people by ICS male questionnaire [4]. Korean study using translated version of ICS male questionnaire also showed that 86 % of male patients with LUTS had the symptom of TD, which was the second most common symptom even though TD has not been regarded as a bothersome symptom [2]. In addition, the reported prevalence of TD in elderly men was less than that of middle aged men [4]. This may imply that TD is an early obstructive symptom and that the patients with TD get used to this symptom as time passes by. Interestingly, our results were consistent with the previous study that the prevalence of TD in male LUTS patients was higher in their 60’s compared with in their 50’s and 70’s. TD does not seem to be the sign of the early stages of LUTS because IPSS total score and IPSS QoL score in TD group were significantly higher than those in non-TD group.

Since there have been few studies investigating TD, its associations with LUTS and several clinical parameters have not been well characterized so far. In this study, TD had an association with voiding subscore in IPSS but no associations with serum PSA level, prostate volume, and prostate transitional zone volume. Traditionally, TD has been regarded as one of the voiding symptoms [4, 6, 13]. In addition, IPP has been reported to be associated with voiding symptoms [9, 20, 21]. In the present study, IPSS voiding subscore had a strong correlation with TD and IPP emerged as a significant risk factor for uroflowmetry-confirmed TD.

Earlier reports have tended to put an emphasis on objective tools such as uroflowmetry. However, 48 % of the patients who reported TD did not show any objective evidence of TD on uroflowmetry [6]. In our study, about 30 % of the patients did not show the typical finding of TD on uroflowmetry. One of the reasons of this result is probably that TD does not happen all the time in some patients. Another reason to be considered is the large amount of urine volume that the patients try to void when they are taking the uroflowmetry test.

This study has several limitations. First, pressure flow studies were not performed, and as a result we could not confirm bladder outlet obstruction of each patient. However, the recent guideline recommends that pressure flow studies should be considered before invasive treatment in men with a Qmax greater than 10 ml per second and are not necessarily needed in the patients whose maximum flow rate is less than 10 ml per second [22]. Second, we did not use the entire ICS male questionnaire because it contains a large number of questions. Notably, however, the initial developers of the questionnaire have recommended the short form ICSmaleSF questionnaire omitting duplicate or redundant items [19].

## Conclusions

Among men with LUTS attending outpatient clinic, about 40 % of them complained of TD. IPSS voiding subscore had a strong correlation with TD and IPP was the only significant risk factor for uroflowmetry-confirmed TD. On the other hand, the parameters such as age, PSA, prostate volume and prostate transitional zone volume did not show any significant association with it. TD is not correlated with each question of IPSS, which means that TD is the specific symptom among several LUTS. Further studies on TD will reveal the clinical impact of this underestimated symptom on LUTS patients, and make it possible to guide a proper management.

## Abbreviations

TD: Terminal dribbling
BPH: Benign prostate hyperplasia
ICS: International continence society
LUTS: Lower urinary tract symptom
IPSS: International prostate symptom score
PSA: Prostate-specific antigen
TRUS: Transrectal ultrasonography
QoL: Quality of life
